# Influence of Autapses on Synchronization in Neural Networks With Chemical Synapses

**DOI:** 10.3389/fnsys.2020.604563

**Published:** 2020-11-30

**Authors:** Paulo R. Protachevicz, Kelly C. Iarosz, Iberê L. Caldas, Chris G. Antonopoulos, Antonio M. Batista, Jurgen Kurths

**Affiliations:** ^1^Institute of Physics, University of São Paulo, São Paulo, Brazil; ^2^Faculdade de Telêmaco Borba, FATEB, Telêmaco Borba, Brazil; ^3^Graduate Program in Chemical Engineering, Federal University of Technology Paraná, Ponta Grossa, Brazil; ^4^Department of Mathematical Sciences, University of Essex, Colchester, United Kingdom; ^5^Department of Mathematics and Statistics, State University of Ponta Grossa, Ponta Grossa, Brazil; ^6^Department Complexity Science, Potsdam Institute for Climate Impact Research, Potsdam, Germany; ^7^Department of Physics, Humboldt University, Berlin, Germany; ^8^Centre for Analysis of Complex Systems, Sechenov First Moscow State Medical University, Moscow, Russia

**Keywords:** synchronization, neural dynamics, integrate-and-fire model, excitatory and inhibitory neural networks, synapses, autapses

## Abstract

A great deal of research has been devoted on the investigation of neural dynamics in various network topologies. However, only a few studies have focused on the influence of autapses, synapses from a neuron onto itself via closed loops, on neural synchronization. Here, we build a random network with adaptive exponential integrate-and-fire neurons coupled with chemical synapses, equipped with autapses, to study the effect of the latter on synchronous behavior. We consider time delay in the conductance of the pre-synaptic neuron for excitatory and inhibitory connections. Interestingly, in neural networks consisting of both excitatory and inhibitory neurons, we uncover that synchronous behavior depends on their synapse type. Our results provide evidence on the synchronous and desynchronous activities that emerge in random neural networks with chemical, inhibitory and excitatory synapses where neurons are equipped with autapses.

## 1. Introduction

An important research subject in neuroscience is to understand how cortical networks avoid or reach states of high synchronization (Kada et al., 2016). In normal activity, excitatory and inhibitory currents are well balanced (Tatti et al., 2018; Zhou and Yu, 2018), while in epileptic seizures, high synchronous behavior has been related to unbalanced current inputs (Drongelen et al., 2005; Avoli et al., 2016). Nazemi and Jamali (2018) showed that the structural coupling strength is important for the appearance of synchronized activities in excitatory and inhibitory neural populations. Various studies discuss the relation between structure and function in microscale and macroscale brain networks (Sporns, 2013a; DeBello et al., 2014; Sporns, 2016; Suárez et al., 2020). In a microscale organization, local excitatory and inhibitory connections are responsible for a wide range of neural interactions (Sporns, 2012; Feng et al., 2018). Bittner et al. (2017) investigated population activity structure as a function of neuron types. They verified that the population activity structure depends on the ratio of excitatory to inhibitory neurons sampled. The pyramidal cell (excitatory neuron) exhibit spike adaptation, while the fast spiking cell (inhibitory neuron) have a small or inexistent spike adaptation (Neske et al., 2015; Descalzo, 2005).

The excitatory to inhibitory and inhibitory to excitatory connections can change firing rates, persistent activities and synchronization of the population of postsynaptic neurons (Börgers and Kopell, 2003; Han et al., 2018; Hayakawa and Fukai, 2020; Kraynyukova and Tchumatchenko, 2018; Mahmud and Vassanelli, 2016). Deco et al. (2014) analyzed the effect of control in the inhibitory to excitatory coupling on the neural firing rate. Mejias et al. (2018) proposed a computational model for the primary cortex in which different layers of excitatory and inhibitory connections were considered.

A number of studies reported that excitatory synapses facilitate neural synchronization (Borges et al., 2017; Breakspear et al., 2003), while inhibitory synapses have an opposite effect (Kada et al., 2016; Ostojic, 2014; Protachevicz et al., 2019). The time delay related to excitatory and inhibitory synapses influences the neural synchronization (Gu and Zhou, 2015; Protachevicz et al., 2020). Further on, there is a strong research interest in the investigation of how excitatory and inhibitory synapses influence synchronization in neural networks (Ge and Cao, 2019). On the other hand, different types of networks have been used to analyse neural synchronization, such as random (Bondarenko and Chay, 1998; Gray and Robinson, 2008), small-world (Antonopoulos et al., 2015, 2016; Hizanidis et al., 2016; Kim and Lim, 2013; Li and Zheng, 2010; Qu et al., 2014), regular (Santos et al., 2019; Wang et al., 2007), and scale-free (Lombardi et al., 2017; Wang et al., 2011).

Experiments showed that autapses are common in the brain and that they play an important role in neural activity (Bekkers, 1998; Pouzat and Marty, 1998; Wang and Chen, 2015). An autapse is a synaptic contact from a neuron to itself via a closed loop (Bekkers, 2009; van der Loos and Glaser, 1972), i.e., an auto-connection with a time delay on signal transmission (Ergin et al., 2016). Although, autaptic connections are anatomically present *in vivo* and in the neocortex, their functions are not completely understood (Bacci et al., 2003). Experimental and theoretical studies on excitatory and inhibitory autapses have been carried out (Tamás et al., 1997; Saada-Madar et al., 2012; Suga et al., 2014; Szegedi et al., 2020) and the results have demonstrated that autaptic connections play a significant role in normal and abnormal brain dynamics (Wyart et al., 2005; Valente et al., 2016; Wang et al., 2017; Yao et al., 2019). The effects of autapses on neural dynamics were studied for single neurons (Heng-Tong and Yong, 2015; Herrmann and Klaus, 2004; Jia, 2018; Kim, 2019) and for neural networks (HuiXin et al., 2014). It has been shown that excitatory autapses contribute to a positive feedback (Zhao and Gu, 2017) and can maintain persistent activities in neurons (Bekkers, 2009). It was also found that they promote burst firing patterns (Wiles et al., 2017; Ke et al., 2019). The inhibitory autapses contribute to a negative feedback (Bacci et al., 2003; Zhao and Gu, 2017) and to the reduction of neural excitability (Bekkers, 2003; Qin et al., 2014; Szegedi et al., 2020). Guo et al. (2016) analyzed chemical and electrical autapses in the regulation of irregular neural firing. In this way, autaptic currents can modulate neural firing rates (Bacci et al., 2003). Wang et al. (2014) demonstrated that chemical autapses can induce a filtering mechanism in random synaptic inputs. Interestingly, inhibitory autapses can favor synchronization during cognitive activities (Deleuze et al., 2019). Short-term memory storage was observed by Seung et al. (2000) in a neuron with autapses submitted to excitatory and inhibitory currents. Finally, a study on epilepsy has exhibited that the number of autaptic connections can be different in her epileptic tissue (Bacci et al., 2003).

Here, we construct a random network with adaptive exponential integrate-and-fire (AEIF) neurons coupled with chemical synapses. The model of AEIF neurons was proposed by Brette and Gerstner (2005) and has been used to mimic neural spike and burst activities. Due to the fact that the chemical synapses can be excitatory and inhibitory, we build a network with excitatory synapses and autapses, a network with inhibitory synapses and autapses, and a network with both types of synapses and autapses. In the mixed network, we consider 80% of excitatory and 20% of inhibitory synapses and autapses. In this work, we focus on the investigation of the influence of autapses on neural synchronization. Ladenbauer et al. (2013) studied the role of adaptation in excitatory and inhibitory populations of AEIF neurons upon synchronization, depending on whether the recurrent synaptic excitatory or inhibitory couplings dominate. In our work, we show that not only the adaptation, but also the autapses can play an important role in the synchronous behavior. To do so, we compute the order parameter to quantify synchronization, the coefficient of variation in neural activity, firing rates and synaptic current inputs. In our simulations, we observe that autapses can increase or decrease synchronous behavior in neural networks with excitatory synapses. However, when only inhibitory synapses are considered, synchronization does not suffer significant alterations in the presence of autapses. Interestingly, in networks with excitatory and inhibitory synapses, we show that excitatory autapses can give rise to synchronous or desynchronous neural activity. Our results provide evidence how synchronous and desynchronous activities can emerge in neural networks due to autapses and contribute to understanding further the relation between autapses and neural synchronization.

The paper is organized as follows: in section 2, we introduce the neural network of AEIF neurons and the diagnostic tools that will be used, such as the order parameter for synchronization, the coefficient of variation, the firing rates and synaptic current inputs. In section 3, we present the results of our study concerning the effects of autapses in neural synchronization, and in section 4, we draw our conclusions.

## 2. Methods

### 2.1. The AEIF Model With Neural Autapses and Network Configurations

The cortex comprises mainly excitatory pyramidal neurons and inhibitory interneurons (Atencio and Schreiner, 2008). Inhibitory neurons have a relatively higher firing rate than excitatory ones (Wilson et al., 1994; Inawashiro et al., 1999; Baeg et al., 2001). In the mammalian cortex, the firing pattern of excitatory neurons corresponds to regular spiking (Neske et al., 2015), while inhibitory neurons exhibit fast spiking activities (Wang et al., 2016). Furthermore, excitatory neurons show adaptation properties in response to depolarizing inputs and the inhibitory adaptation current is negligible or nonexistent (Foehring et al., 1991; Mancilla et al., 1998; Hensch and Fagiolini, 2004; Destexhe, 2009; Masia et al., 2018; Borges et al., 2020). The fast spiking interneurons are the most common inhibitory neurons in the cortex (Puig et al., 2008).

In the neural networks considered in this work, the dynamics of each neuron *j*, where *j* = 1, …, *N*, is given by the adaptive exponential integrate-and-fire model. In this framework, *N* denotes the total number of neurons in the network. The AEIF model is able to reproduce different firing patterns, including regular and fast spiking (di Volo et al., 2019). The network dynamics is given by the following set of coupled, nonlinear, ordinary differential equations

(1) $$\begin{array}{l} C_{\text{m}}\frac{dV_{j}}{dt}=-g_{\text{L}}\left(V_{j}-E_{\text{L}}\right)+g_{\text{L}}\Delta_{\text{T}}\text{exp}\left(\frac{V_{j}-V_{\text{T}}}{\Delta_{\text{T}}}\right)\\ \;-w_{j}+I+I_{j}^{\text{chem}}\left(t\right),\\ \begin{array}{l} \;\tau_{w}\frac{dw_{j}}{dt}=a_{j}\left(V_{j}-E_{\text{L}}\right)-w_{j}, \end{array}\\ \begin{array}{l} \;\tau_{\text{s}}\frac{dg_{j}}{dt}=-g_{j}, \end{array} \end{array}$$

where *V*_*j*_ is the membrane potential, *w*_*j*_ the adaptation current and *g*_*j*_ the synaptic conductance of neuron *j*. *k* and *j* identify the pre and postsynaptic neurons. When the membrane potential of neuron *j* is above the threshold *V*_thres_, i.e., when *V*_*j*_ > *V*_thres_ (Naud et al., 2008), the state variables are updated according to the rules

(2) $$\begin{array}{l} \;V_{j}\to V_{\text{r}},\\ \begin{array}{l} w_{j}\to w_{j}+b_{j}, \end{array}\\ \begin{array}{l} \;g_{j}\to g_{j}+g_{\text{s}}, \end{array} \end{array}$$

where *g*_s_ assumes the value $g_{\text{e}}^{\text{aut}}$ for excitatory autapses, *g*_e_ for synapses among excitatory neurons, *g*_ei_ for synapses from excitatory to inhibitory neurons, $g_{\text{i}}^{\text{aut}}$ for inhibitory autapses, *g*_i_ for synapses among inhibitory neurons and *g*_ie_ for synapses from inhibitory to excitatory neurons. We consider a neuron is excitatory (inhibitory) when it is connected to another neuron with an excitatory (inhibitory) synapse. The initial conditions of *V*_*j*_ are randomly distributed in the interval *V*_*j*_ = [−70, −50] mV. The initial values of *w*_*j*_ are randomly distributed in the interval *w*_*j*_ = [0, 300] pA for excitatory and *w*_*j*_ = [0, 80] pA for inhibitory neurons. We consider the initial value of *g*_*j*_ equal to zero for all neurons. Table 1 summarizes the description and values of the parameters used in the simulations.

**Table 1:** Description and values of the parameters in the AEIF system (1) and (2) used in the simulations.

**Parameter**	**Description**	**Value**
*N*	Number of AEIF neurons	1,000 neurons
*C*_m_	Membrane capacitance	200 pF
*g*_L_	Leak conductance	12 nS
*E*_L_	Leak reversal potential	−70 mV
*I*	Constant input current	270 pA
Δ_T_	Slope factor	2 mV
*V*_T_	Potential threshold	−50 mV
τ_*w*_	Adaptation time constant	300 ms
τ_s_	Synaptic time constant	2.728 ms
*V*_r_	Reset potential	−58 mV
$M_{jk}^{\text{exc}}$	Adjacency matrix elements	0 or 1
$M_{jk}^{\text{inh}}$	Adjacency matrix elements	0 or 1
*t*_ini_	Initial time in the analyses	10 s
*t*_fin_	Final time in the analyses	20 s
*a*_*j*_	Subthreshold adaptation	[1.9, 2.1] nS •
		0 nS ⋆
*b*_*j*_	Triggered adaptation	70 pA •
		0 pA ⋆
*V*_REV_	Synaptic reversal potential	$V_{\text{REV}}^{\text{exc}}=0$ mV •
		$V_{\text{REV}}^{\text{inh}}=$-80 mV ⋆
*g*_s_	Chemical conductances	*g*_e_, $g_{\text{e}}^{\text{aut}}$, *g*_ei_ •
		*g*_i_, $g_{\text{i}}^{\text{aut}}$, *g*_ie_ ⋆
*g*_e_	Excitatory to excitatory	[0,0.5] nS •
$g_{\text{e}}^{\text{aut}}$	Excitatory autaptic	[0,35] nS •
*g*_ei_	Excitatory to inhibitory	[0,5] nS •
*g*_i_	Inhibitory to inhibitory	[0,2] nS ⋆
$g_{\text{i}}^{\text{aut}}$	Inhibitory autaptic	[0,100] nS ⋆
*g*_ie_	Inhibitory to excitatory	[0,3] nS ⋆
*d*_*j*_	Time delay	*d*_exc_ = 1.5 ms •
		*d*_inh_ = 0.8 ms ⋆

*Values for parameters for excitatory and inhibitory connections are denoted by • and ⋆, respectively*.

The synaptic current arriving at each neuron depends on specific parameters, including the connectivity encoded in the adjacency matrices *M*^exc^ and *M*^inh^, i.e., in the excitatory and inhibitory connectivity matrices. In particular, the input current $I_{j}^{\text{chem}}$ arriving at each neuron *j*, is calculated by

(3) $$\begin{array}{l} I_{j}^{\text{chem}}\left(t\right)=I_{j}^{\text{exc}}\left(t\right)+I_{j}^{\text{inh}}\left(t\right), \end{array}$$

where

$$\begin{array}{l} I_{j}^{\text{exc}}\left(t\right)=I_{j}^{\text{ee}}\left(t\right)+I_{j}^{\text{ei}}\left(t\right)+I_{j}^{\text{e},\text{aut}}\left(t\right)\\ \;=\left(V_{\text{REV}}^{\text{exc}}-V_{j}\left(t\right)\right)\overset{N}{\underset{k=1}{\sum }}M_{jk}^{\text{exc}}g_{k}\left(t-d_{\text{exc}}\right) \end{array}$$

and

$$\begin{array}{l} I_{j}^{\text{inh}}\left(t\right)=I_{j}^{\text{ii}}\left(t\right)+I_{j}^{\text{ie}}\left(t\right)+I_{j}^{\text{i},\text{aut}}\left(t\right)\\ \;=\left(V_{\text{REV}}^{\text{inh}}-V_{j}\left(t\right)\right)\overset{N}{\underset{k=1}{\sum }}M_{jk}^{\text{inh}}g_{k}\left(t-d_{\text{inh}}\right). \end{array}$$

In this framework, the type of synapse (excitatory or inhibitory) depends on the synaptic reversal potential *V*_REV_. We consider $V_{\text{REV}}^{\text{exc}}=0$ mV for excitatory and $V_{\text{REV}}^{\text{inh}}=-80$ mV for inhibitory synapses. The time delay in the conductance of the pre-synaptic neuron *k* (*g*_*k*_) assumes *d*_exc_ = 1.5 ms for excitatory and *d*_inh_ = 0.8 ms for inhibitory connections (Borges et al., 2020). The influence of delayed conductance on neural synchronization was studied in Protachevicz et al. (2020). There are no spike activities in the time interval *t* = [−*d*_*j*_, 0].

**Figure 1:**
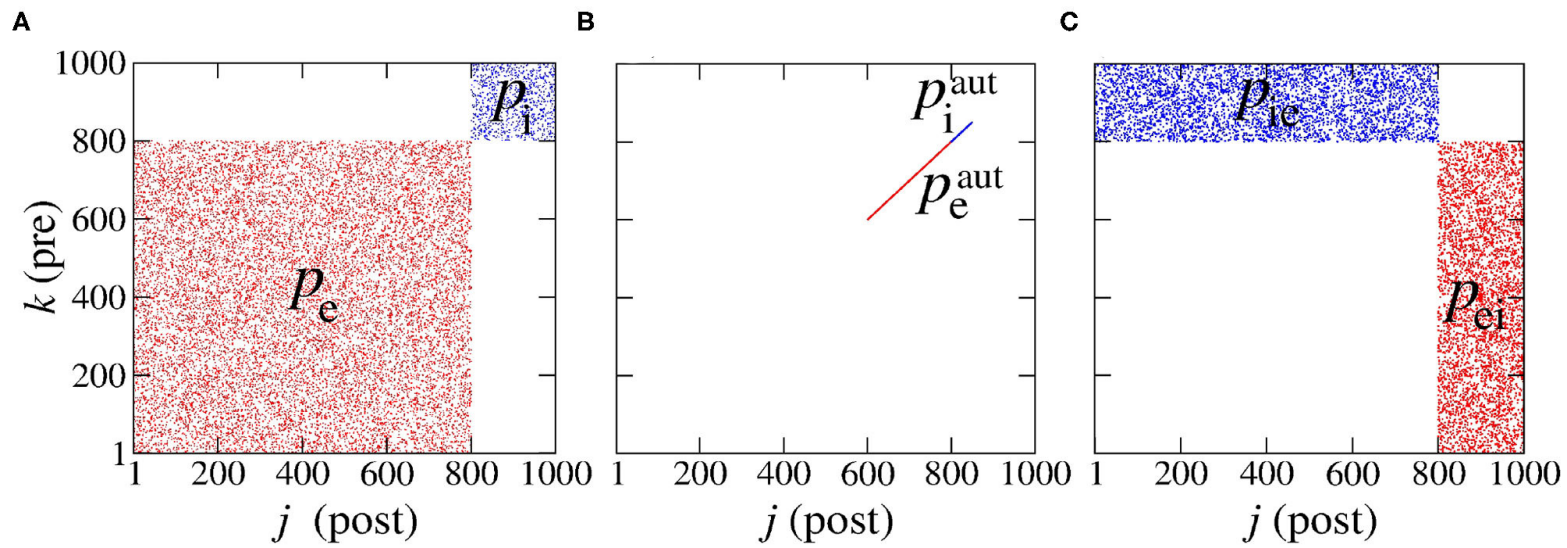
Representation of the connections: **(A)** in the same population, **(B)** for autapses, and **(C)** among different neural populations. Here, “pre” stands for “pre-synaptic” and “post” for “post-synaptic.” We note that we have used *P*_exc_ = 80% excitatory (denoted red) and *P*_inh_ = 20% inhibitory (denoted blue) neural populations which amounts to a total of *N* = 1,000 neurons.

The first *N*_exc_ neurons are excitatory and the last *N*_inh_ inhibitory. The connections that depart from excitatory and inhibitory neurons are associated with the excitatory and inhibitory matrices, *M*^exc^ and *M*^inh^, where each entry is denoted $M_{jk}^{\text{exc}}$ and $M_{jk}^{\text{inh}}$, respectively. These adjacency matrices are binary and have entries equal to 1 when there is a connection from neuron *k* to neuron *j*, or 0 otherwise, as shown in Figure 1.

We consider *P*_exc_ = 80% excitatory and *P*_inh_ = 20% inhibitory neural populations following di Volo et al. (2019) and Noback et al. (2005), where the numbers of excitatory and inhibitory neurons are given by *N*_exc_ = *P*_exc_*N* and *N*_inh_ = *P*_inh_*N*, respectively. The connectivity probabilities are set to $p_{\text{e}}^{\text{aut}}=p_{\text{i}}^{\text{aut}}=0.25$ for excitatory and inhibitory autapses, to *p*_e_ = 0.05 and *p*_i_ = 0.2 for connectivity within the same neural population and to *p*_ei_ = *p*_ie_ = 0.05 for connectivity among different neural populations (di Volo et al., 2019). The subscripts “e” and “i” stand for “excitatory” and “inhibitory”, respectively and the superscript “aut” stands for “autapses.” The terms *p*_ei_ and *p*_ie_ represent the probabilities of connections from excitatory to inhibitory and from inhibitory to excitatory neurons, respectively.

The probabilities of excitatory and inhibitory autapses are defined by

$$\begin{array}{l} p_{\text{e}}^{\text{aut}}=\frac{N_{\text{e}}^{\text{aut}}}{N_{\text{exc}}}\text{ and }p_{\text{i}}^{\text{aut}}=\frac{N_{\text{i}}^{\text{aut}}}{N_{\text{inh}}}, \end{array}$$

where $N_{\text{e}}^{\text{aut}}$ and $N_{\text{i}}^{\text{aut}}$ are the number of autapses in the excitatory and inhibitory populations, respectively. For a network with only excitatory (inhibitory) neurons, the number of excitatory (inhibitory) neurons is *N*_exc_ = *N* (*N*_inh_ = *N*). For connections within the excitatory and inhibitory populations, the corresponding probabilities *p*_e_ and *p*_i_ are given by

$$\begin{array}{l} p_{\text{e}}=\frac{N_{\text{e}}}{N_{\text{exc}}\left(N_{\text{exc}}-1\right)}\text{ and }p_{\text{i}}=\frac{N_{\text{i}}}{N_{\text{inh}}\left(N_{\text{inh}}-1\right)}, \end{array}$$

where *N*_e_ and *N*_i_ are the number of synaptic connections in the excitatory and inhibitory populations, respectively. For connections among different populations, the corresponding probabilities are given by

$$\begin{array}{l} p_{\text{ei}}=\frac{N_{\text{ei}}}{N_{\text{exc}}N_{\text{inh}}}\text{ and }p_{\text{ie}}=\frac{N_{\text{ie}}}{N_{\text{exc}}N_{\text{inh}}}, \end{array}$$

where *N*_ei_ and *N*_ie_ are the number of synaptic connections from the excitatory to the inhibitory and from the inhibitory to the excitatory populations, respectively. Therefore, when only one neural population is considered, *p*_ei_ and *p*_ie_ cannot be defined. The resulting six connectivity probabilities are represented in the connectivity matrix in Figure 1, where *k* and *j* denote the pre- and post-synaptic neurons, respectively. Figure 1 shows the connections associated to probabilities: (Figure 1A) in the same population (*p*_e_ and *p*_i_), (Figure 1B) for autapses ($p_{\text{e}}^{\text{aut}}$ and $p_{\text{i}}^{\text{aut}}$) and (Figure 1C) among different populations (*p*_ei_ and *p*_ie_).

Finally, we associate the conductances *g*_e_, *g*_i_, $g_{\text{e}}^{\text{aut}}$, $g_{\text{i}}^{\text{aut}}$, *g*_ei_, and *g*_ie_ to the corresponding connectivity probabilities discussed before. To solve the set of ordinary differential equations in system (1), we used the 4th order Runge-Kutta method with the integration time-step equal to 10^−2^ ms.

### 2.2. Computation of Neural Synchronization

Synchronous behavior in neural networks can be quantified by means of the order parameter *R* (Kuramoto, 1984)

$$\begin{array}{l} R\left(t\right)=\left|\frac{1}{N}\overset{N}{\underset{j=1}{\sum }}\text{exp}\left(\text{i}\psi_{j}\left(t\right)\right)\right|, \end{array}$$

where *R*(*t*) is the amplitude of a centroid phase vector over time, i the imaginary unit, satisfying i^2^ = −1, and |·|, the vector-norm of the argument. The phase of each neuron *j* in time is obtained by means of

(4) $$\begin{array}{l} \psi_{j}\left(t\right)=2\pi m+2\pi \frac{t-t_{j,m}}{t_{j,m+1}-t_{j,m}}, \end{array}$$

where *t*_*j,m*_ is the time of the *m*-th spike of neuron *j*, where *t*_*j,m*_ < *t* < *t*_*j, m*+1_ (Rosenblum et al., 1997). We consider that spikes occur whenever *V*_*j*_ > *V*_thres_ (Naud et al., 2008). *R*(*t*) takes values in [0, 1] and, is equal to 0 for completely desynchronized neural activity and 1 for fully synchronized neural behavior. We compute the time-average order parameter $\bar{R}$ (Batista et al., 2017), given by

(5) $$\begin{array}{l} \bar{R}=\frac{1}{t_{\text{fin}}-t_{\text{ini}}}\int_{t_{\text{ini}}}^{t_{\text{fin}}}R\left(t\right)dt, \end{array}$$

where (*t*_fin_ − *t*_ini_) is the length of the time window [*t*_ini_, *t*_fin_]. Here, we have used *t*_ini_ = 10 s and *t*_fin_ = 20 s. Similarly, we calculate the synchronization of the non-autaptic neurons

$$\begin{array}{l} R_{\text{non}}\left(t\right)=\left|\frac{1}{N^{\text{non}}}\overset{N^{\text{non}}}{\underset{j=1}{\sum }}\text{exp}\left(\text{i}\psi_{j}^{\text{non}}\left(t\right)\right)\right| \end{array}$$

and autaptic neurons

$$\begin{array}{l} R_{\text{aut}}\left(t\right)=\left|\frac{1}{N^{\text{aut}}}\overset{N^{\text{aut}}}{\underset{j=1}{\sum }}\text{exp}\left(\text{i}\psi_{j}^{\text{aut}}\left(t\right)\right)\right|, \end{array}$$

where *N*^non^ and *N*^aut^ are the number of non-autaptic and autaptic neurons, respectively. In this context, $\psi_{j}^{\text{non}}$ and $\psi_{j}^{\text{aut}}$ are the phases of the non-autaptic and autaptic neuron *j* and both terms are computed using Equation (4) for the times of spiking of the non-autaptic and autaptic neurons, respectively. ${\bar{R}}_{\text{non}}$ and ${\bar{R}}_{\text{aut}}$ are then obtained according to Equation (5).

### 2.3. Mean Coefficient of Variation of Interspike Intervals

We calculate the interspike intervals of each neuron to obtain the mean coefficient of variation. In particular, the *m*-th interspike interval of neuron *j*, ${\text{ISI}}_{j}^{m}$, is defined as the difference between two consecutive spikes,

$$\begin{array}{l} {\text{ISI}}_{j}^{m}=t_{j,m+1}-t_{j,m}>0, \end{array}$$

where *t*_*j,m*_ is the time of the *m*-th spike of neuron *j*. Using the mean value of ISI_*j*_ over all *m*, ${\bar{\text{ISI}}}_{j}$ and its standard deviation σ_ISI_*j*__, we can compute the coefficient of variation (CV) of neuron *j*,

$$\begin{array}{l} {\text{CV}}_{j}=\frac{\sigma_{{\text{ISI}}_{j}}}{{\bar{\text{ISI}}}_{j}}. \end{array}$$

The average CV over all neurons in the network, $\bar{\text{CV}}$, can then be computed by

$$\begin{array}{l} \bar{\text{CV}}=\frac{1}{N}\overset{N}{\underset{j=1}{\sum }}{\text{CV}}_{j}. \end{array}$$

We use the value of $\bar{\text{CV}}$ to identify spikes whenever $\bar{\text{CV}}<0.5$ and burst firing patterns whenever $\bar{\text{CV}}\geq 0.5$ (Borges et al., 2017; Protachevicz et al., 2018) in neural activity.

### 2.4. Firing Rates in Neural Populations

The mean firing-rate of all neurons in a network is computed by means of

$$\begin{array}{l} \bar{F}=\frac{1}{N\left(t_{\text{fin}}-t_{\text{ini}}\right)}\overset{N}{\underset{j=1}{\sum }}\left(\int_{t_{\text{ini}}}^{t_{\text{fin}}}\delta \left(t^{\prime }-t_{j}\right)dt^{\prime }\right), \end{array}$$

where *t*_*j*_ is the firing time of neuron *j*. In some occasions, we calculate the mean firing frequency of neurons with and without autapses,

$$\begin{array}{l} {\bar{F}}_{\text{aut}}=\frac{1}{N_{\text{x}}^{\text{aut}}\left(t_{\text{fin}}-t_{\text{ini}}\right)}\overset{N_{\text{x}}^{\text{aut}}}{\underset{j=1}{\sum }}\left(\int_{t_{\text{ini}}}^{t_{\text{fin}}}\delta \left(t^{\prime }-t_{j}^{\text{aut}}\right)dt^{\prime }\right) \end{array}$$

and

$$\begin{array}{l} {\bar{F}}_{\text{non}}=\frac{1}{N_{\text{x}}^{\text{non}}\left(t_{\text{fin}}-t_{\text{ini}}\right)}\overset{N_{\text{x}}^{\text{non}}}{\underset{j=1}{\sum }}\left(\int_{t_{\text{ini}}}^{t_{\text{fin}}}\delta \left(t^{\prime }-t_{j}^{\text{non}}\right)dt^{\prime }\right), \end{array}$$

where $N_{\text{x}}^{\text{aut}}$ and $N_{\text{x}}^{\text{non}}$ are the number of neurons with and without autapses, and $t_{j}^{\text{aut}}$ and $t_{j}^{\text{non}}$ the firing times of neurons with and without autapses. The subscript “x” denotes the population of excitatory (“e”) or inhibitory (“i”) neurons.

Similarly, we calculate the firing rate of excitatory and inhibitory neurons by means of

$$\begin{array}{l} {\bar{F}}_{\text{exc}}=\frac{1}{N_{\text{exc}}\left(t_{\text{fin}}-t_{\text{ini}}\right)}\overset{N_{\text{exc}}}{\underset{j=1}{\sum }}\left(\int_{t_{\text{ini}}}^{t_{\text{fin}}}\delta \left(t^{\prime }-t_{j}^{\text{exc}}\right)dt^{\prime }\right) \end{array}$$

and

$$\begin{array}{l} {\bar{F}}_{\text{inh}}=\frac{1}{N_{\text{inh}}\left(t_{\text{fin}}-t_{\text{ini}}\right)}\overset{N_{\text{inh}}}{\underset{j=1}{\sum }}\left(\int_{t_{\text{ini}}}^{t_{\text{fin}}}\delta \left(t^{\prime }-t_{j}^{\text{inh}}\right)dt^{\prime }\right), \end{array}$$

where $t_{j}^{\text{exc}}$ and $t_{j}^{\text{inh}}$ are the firing times of the excitatory and inhibitory neurons, respectively.

### 2.5. Synaptic Current Inputs

In our work, we calculate the mean instantaneous input *I*^chem^(*t*) and the time average of the synaptic input ${\bar{I}}_{\text{s}}$ (pA) in the network by

$$\begin{array}{l} I^{\text{chem}}\left(t\right)=\frac{1}{N}\overset{N}{\underset{j=1}{\sum }}I_{j}^{\text{chem}}\left(t\right) \end{array}$$

and

$$\begin{array}{l} {\bar{I}}_{\text{s}}=\frac{1}{t_{\text{fin}}-t_{\text{ini}}}\int_{t_{\text{ini}}}^{t_{\text{fin}}}I^{\text{chem}}\left(t\right)dt, \end{array}$$

respectively, where $I_{j}^{\text{chem}}\left(t\right)$ is given by Equation (3). In this respect, the values of *I*^chem^ change over time due to excitatory and inhibitory inputs received by neuron *j*, where *j* = 1, …, *N*.

## 3. Results and Discussion

### 3.1. Network With Excitatory Neurons Only

Networks with excitatory neurons were studied previously by Borges et al. (2017) and Protachevicz et al. (2019). These studies showed that excitatory neurons can change firing patterns and improve neural synchronization. Fardet et al. (2018) and Yin et al. (2018) reported that excitatory autapses with few milliseconds time delay can change neural activities from spikes to bursts. Wiles et al. (2017) demonstrated that excitatory autaptic connections contribute more to bursting firing patterns than inhibitory ones.

**Figure 2:**
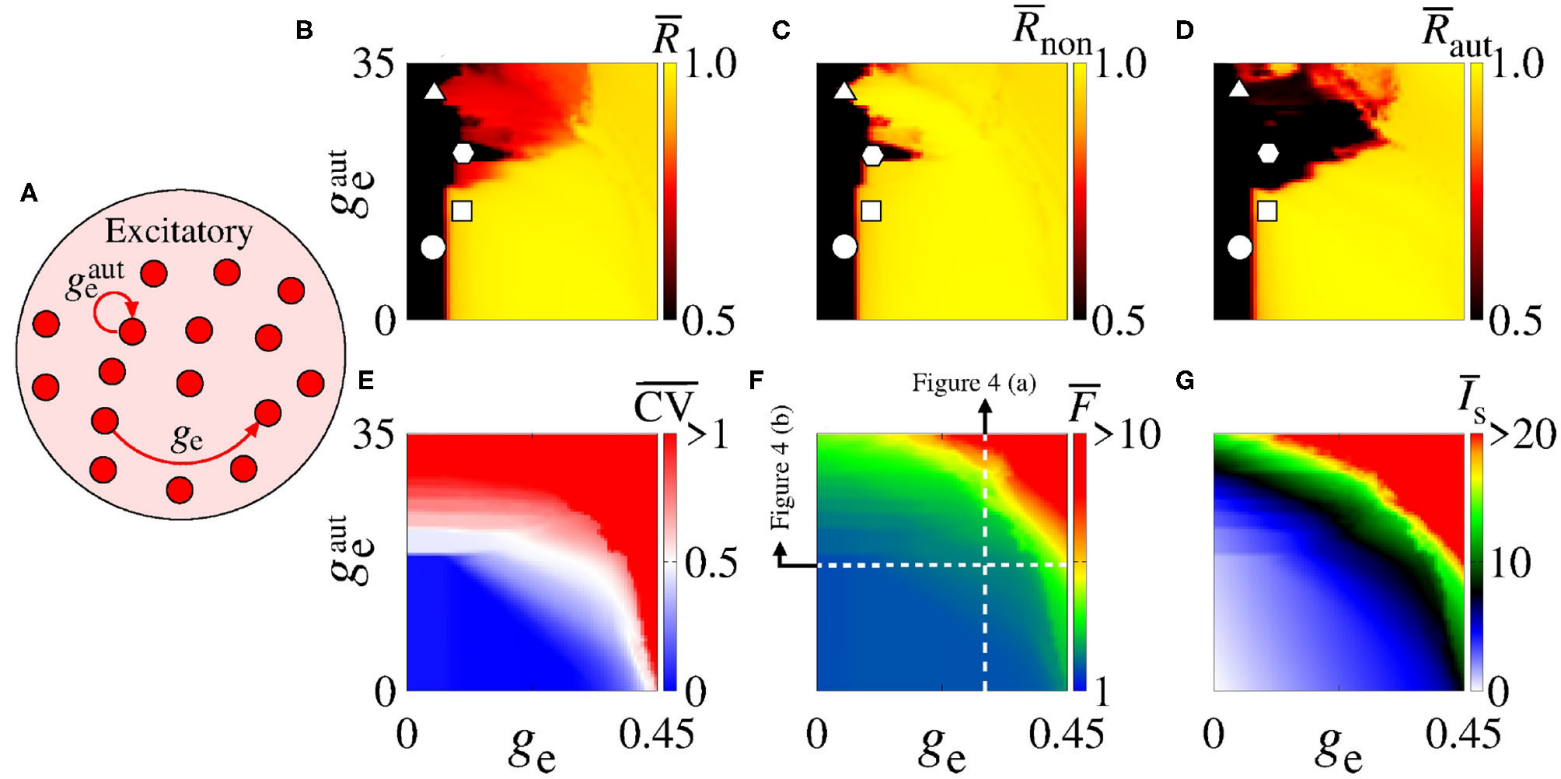
**(A)** Schematic representation of the neural network where *g*_e_ is the intensity of excitatory synaptic conductance and $g_{\text{e}}^{\text{aut}}$ of the excitatory autaptic conductance. Parameter space $g_{\text{e}}\times g_{\text{e}}^{\text{aut}}$, where the color bars correspond to **(B)** $\bar{R}$, **(C)** ${\bar{R}}_{\text{non}}$, **(D)** ${\bar{R}}_{\text{aut}}$, **(E)** $\bar{\text{CV}}$, **(F)** $\bar{F}$, and **(G)** ${\bar{I}}_{\text{s}}$. The raster plots of the parameters indicated in **(B–D)** (circle, square, triangle, and hexagon) are shown in Figure 3. The vertical and horizontal white, dash, lines in **(F)** are used to vary $g_{\text{e}}^{\text{aut}}$ and *g*_e_ in the computations in Figures 4A,B, respectively. The closed loop in **(A)** corresponds to an autapse of excitatory autaptic conductance $g_{\text{e}}^{\text{aut}}$.

**Figure 3:**
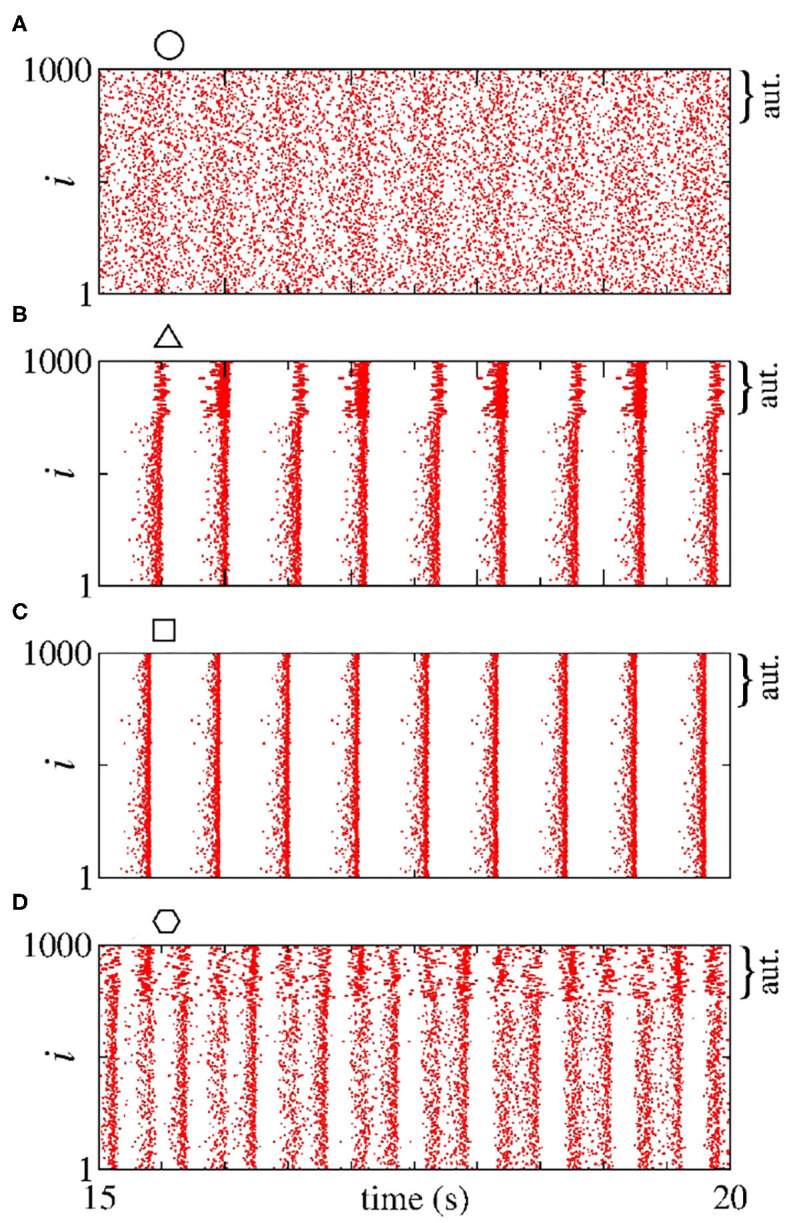
**(A–D)** Raster plots for the neural network with excitatory neurons only. The values of the parameters *g*_e_ and $g_{\text{e}}^{\text{aut}}$ are indicated in Figures 2B–D by circle, triangle, square, and hexagon symbols, respectively. The curly brackets in the upper right corner of the plots denote the autaptic neurons considered.

In Figure 2, we consider a neural network with excitatory neurons only, where *g*_e_ corresponds to the intensity of excitatory synaptic conductance and $g_{\text{e}}^{\text{aut}}$ to the intensity of excitatory autaptic conductance. In our neural network, a neuron receives many connections from other neurons with small intensity of synaptic conductances. For the autaptic neurons, only one synaptic contact from a neuron to itself via a closed loop is considered. Due to this fact, to study the autaptic influence on the high synchronous activities, we consider values of $g_{\text{e}}^{\text{aut}}$ greater than *g*_e_. Figure 2A shows a schematic representation of a neural network of excitatory neurons only with a single autapse represented by the closed loop with excitatory autaptic conductance $g_{\text{e}}^{\text{aut}}$. Figures 2B–D give the mean order parameter in the parameter space $g_{\text{e}}\times g_{\text{e}}^{\text{aut}}$. We see that excitatory autapses can increase or reduce the synchronization in a population of excitatory neurons when the intensity of the excitatory synaptic conductance is small. In these panels, the circle (*g*_e_ = 0.05 nS and $g_{\text{e}}^{\text{aut}}=10$ nS), triangle (*g*_e_ = 0.05 nS and $g_{\text{e}}^{\text{aut}}=31$ nS), square (*g*_e_ = 0.1 nS and $g_{\text{e}}^{\text{aut}}=15$ nS), and hexagon (*g*_e_ = 0.1 nS and $g_{\text{e}}^{\text{aut}}=22$ nS) symbols indicate the values of the parameters shown in Figure 3. We observe that desynchronous firing patterns as seen in Figure 3A can become more synchronous, as it can be seen in Figure 3B, due the increase of the excitatory autaptic conductance. On the other hand, the increase of the autaptic conductance can decrease the level of synchronization in the network, i.e., from high in Figure 3C to low synchronous activities in Figure 3D. However, as shown in Figure 2D, the autaptic connections affect mainly the synchronization of autaptic neurons.

For a strong excitatory synaptic coupling (*g*_e_ ≥ 0.3), autapses do not reduce neural synchronization significantly. Figures 2E–G show the mean coefficient of variation ($\bar{\text{CV}}$), firing frequency ($\bar{F}$), and synaptic current (${\bar{I}}_{\text{s}}$), respectively. We verify that the excitatory autaptic neurons promote the increase of $\bar{\text{CV}}$, $\bar{F}$ and ${\bar{I}}_{\text{s}}$ in the network. In Figure 2E, we find that both synaptic and autaptic couplings can lead to burst activities, as reported by Borges et al. (2017) and Fardet et al. (2018). The burst and spike activities are characterized by $\bar{\text{CV}}\geq 0.5$ (red region) and $\bar{\text{CV}}<0.5$ (blue region), respectively. In addition, excitatory autaptic neurons can change the firing patterns of all neurons in the network from spike to burst activities. In Figures 2F,G, we observe that excitatory autapses contribute to the increase of the mean firing frequency and synaptic current.

**Figure 4:**
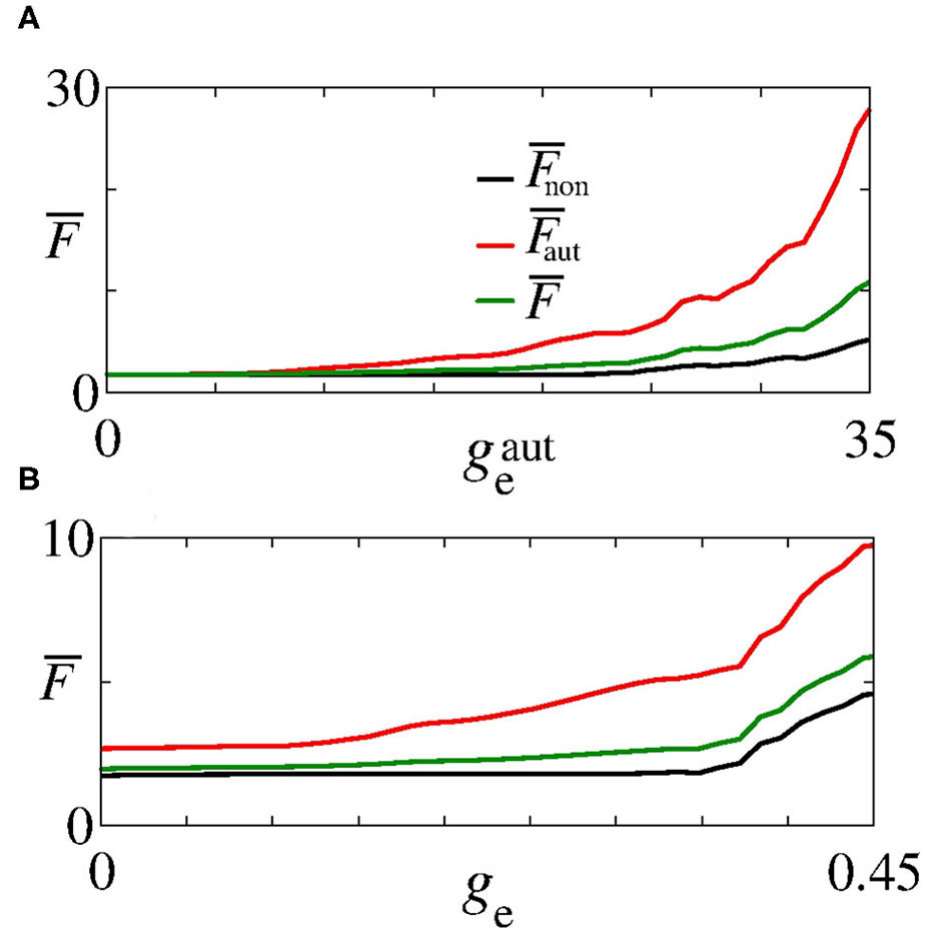
Plot of ${\bar{F}}_{\text{non}}$ (black curve), ${\bar{F}}_{\text{aut}}$ (red curve), and $\bar{F}$ (green curve) for **(A)** *g*_e_ = 0.3 nS varying $g_{\text{e}}^{\text{aut}}$ and **(B)** $g_{\text{e}}^{\text{aut}}=20$ nS varying *g*_e_. Here, $g_{\text{e}}^{\text{aut}}$ and *g*_e_ vary along the white, dash, lines in Figure 2F.

Next, we analyse the influence of autaptic connections on neural firing frequency. Figure 4 shows the mean firing frequency of neurons without (${\bar{F}}_{\text{non}}$) and with autapses (${\bar{F}}_{\text{aut}}$), as well as of all neurons in the excitatory network ($\bar{F}$). In Figure 4A, we consider *g*_e_ = 0.3 nS varying $g_{\text{e}}^{\text{aut}}$, while in Figure 4B, we use $g_{\text{e}}^{\text{aut}}=20$ nS varying *g*_e_, as shown in Figure 2F with white, dash, lines. We find that the autaptic connections increase the firing frequency of all neurons in the network and mainly those with autaptic connections. In our simulations, neurons with excitatory autapses exhibit the highest firing rate.

### 3.2. Network With Inhibitory Neurons Only

**Figure 5:**
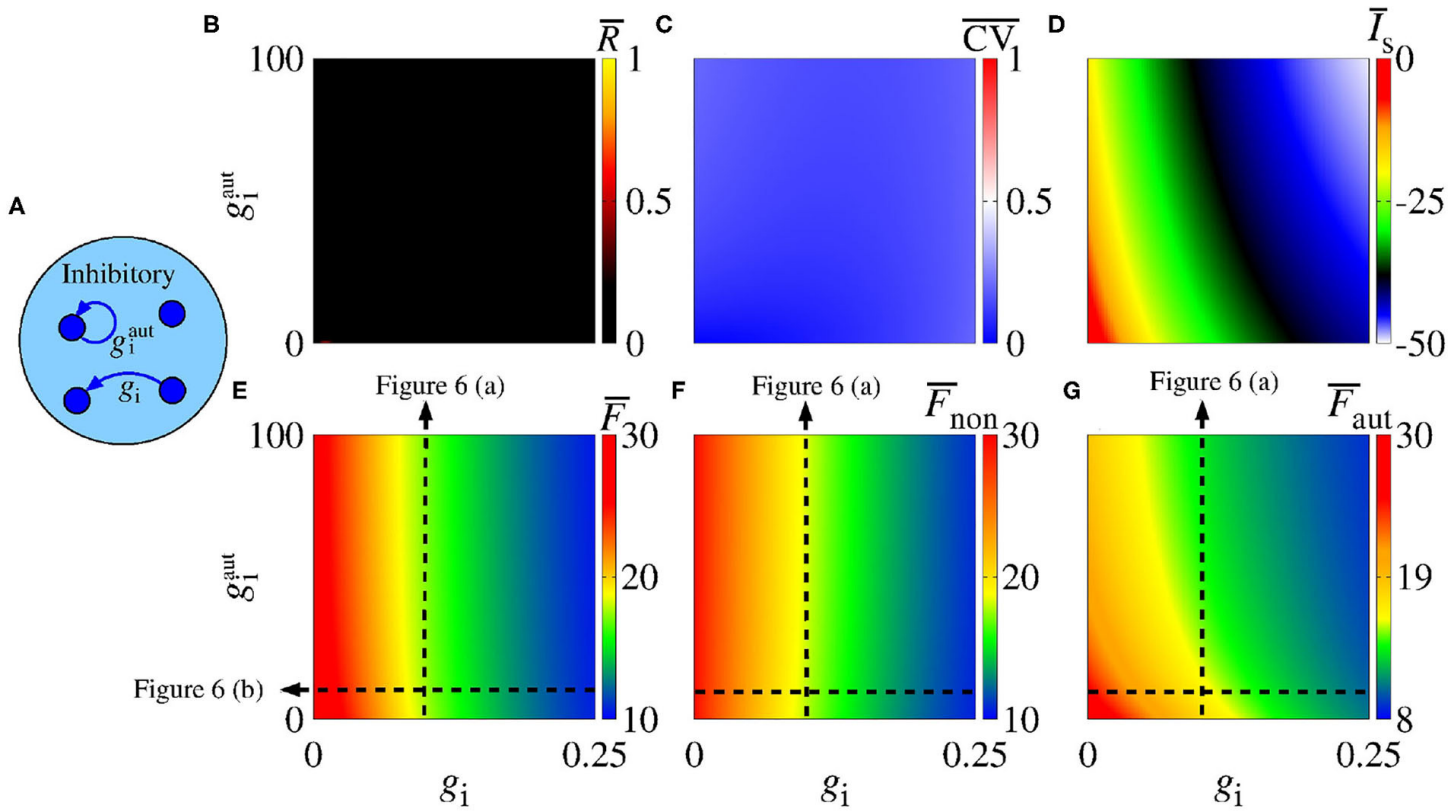
**(A)** Schematic representation of an inhibitory neural population connected with inhibitory synapses and autapses. Parameter space $g_{\text{i}}\times g_{\text{i}}^{\text{aut}}$, where the color bars encode the values of **(B)** $\bar{R}$, **(C)** $\bar{\text{CV}}$, **(D)** ${\bar{I}}_{\text{s}}$, **(E)** $\bar{F}$, **(F)** ${\bar{F}}_{\text{non}}$, and **(G)** ${\bar{F}}_{\text{aut}}$. The vertical and horizontal black, dash, lines in **(E–G)** are used to vary the corresponding parameters in the computations in Figures 6A,B. The closed loop in **(A)** corresponds to an autapse of conductance intensity $g_{\text{i}}^{\text{aut}}$.

Synaptic inhibition regulates the level of neural activity and can prevent hyper excitability (Fröhlich, 2016). Studies have shown that neural networks can exhibit synchronous activities due to inhibitory synapses (van Vreeswijk et al., 1994; Elson et al., 2002; Franović and Miljković, 2010; Chauhan et al., 2018). Here, we analyse the influence of inhibitory synapses and autapses by varying *g*_i_ and $g_{\text{i}}^{\text{aut}}$, as shown in Figure 5A. Figure 5B shows that inhibitory synapses and autapses do not give rise to the increase of neural synchronization in the network. Actually, neural synchronization due to inhibition is possible when it is considered together with other mechanisms related to neural interactions (Bartos et al., 2002), e.g., with gap junctions associated to inhibitory synapses (Bou-Flores and Berger, 2000; Beierlein et al., 2000; Kopell and Ermentrout, 2004; Bartos et al., 2007; Pfeuty et al., 2007; Guo et al., 2012; Reimbayev et al., 2017).

In our simulations, we do not observe that inhibitory interactions promote synchronization in the network. Although this is not surprising, it helps to identify the role of inhibitory autapses in neural synchronization. Figure 5C shows that there is no change from spike to burst patterns, either. In Figure 5D, we verify that both inhibitory synapses and autapses increase the intensity of the mean negative synaptic current.

**Figure 6:**
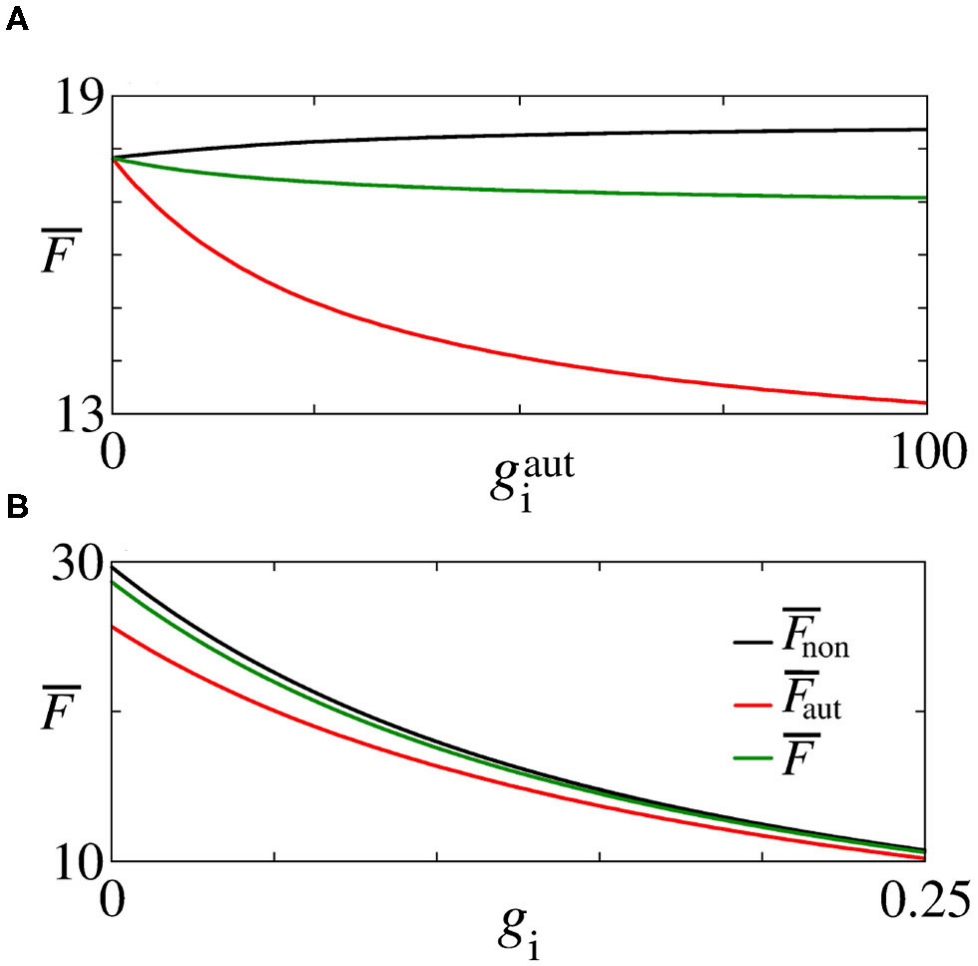
Plot of ${\bar{F}}_{\text{non}}$ (black line), ${\bar{F}}_{\text{aut}}$ (red line), and $\bar{F}$ (green line) for **(A)** *g*_i_ = 0.1 nS varying $g_{\text{i}}^{\text{aut}}$ and **(B)** $g_{\text{i}}^{\text{aut}}=10$ nS varying *g*_i_, indicated in Figure 5 by the black, dash, lines.

In Figure 5E, we see that inhibitory synapses contribute to the decrease of $\bar{F}$, while Figures 5F,G show the mean firing rate for non-autaptic neurons, i.e., neurons without autapses (${\bar{F}}_{\text{non}}$) and for autaptic neurons (${\bar{F}}_{\text{aut}}$), respectively. The autapses reduce the firing-rate of the autaptic neurons, what can lead to an increase of the firing rate of the non-autaptic neurons. This can be better observed in Figure 6A, which shows the values of ${\bar{F}}_{\text{non}}$, ${\bar{F}}_{\text{aut}}$, and $\bar{F}$ as a function of $g_{\text{i}}^{\text{aut}}$ for *g*_i_ = 0.1 nS. Figure 6B shows the mean firing rates as a function of *g*_i_ for $g_{\text{i}}^{\text{aut}}=10$ nS. The neurons with inhibitory autapses have lower firing rates.

### 3.3. Network With a Mix of Excitatory and Inhibitory Neurons

**Figure 7:**
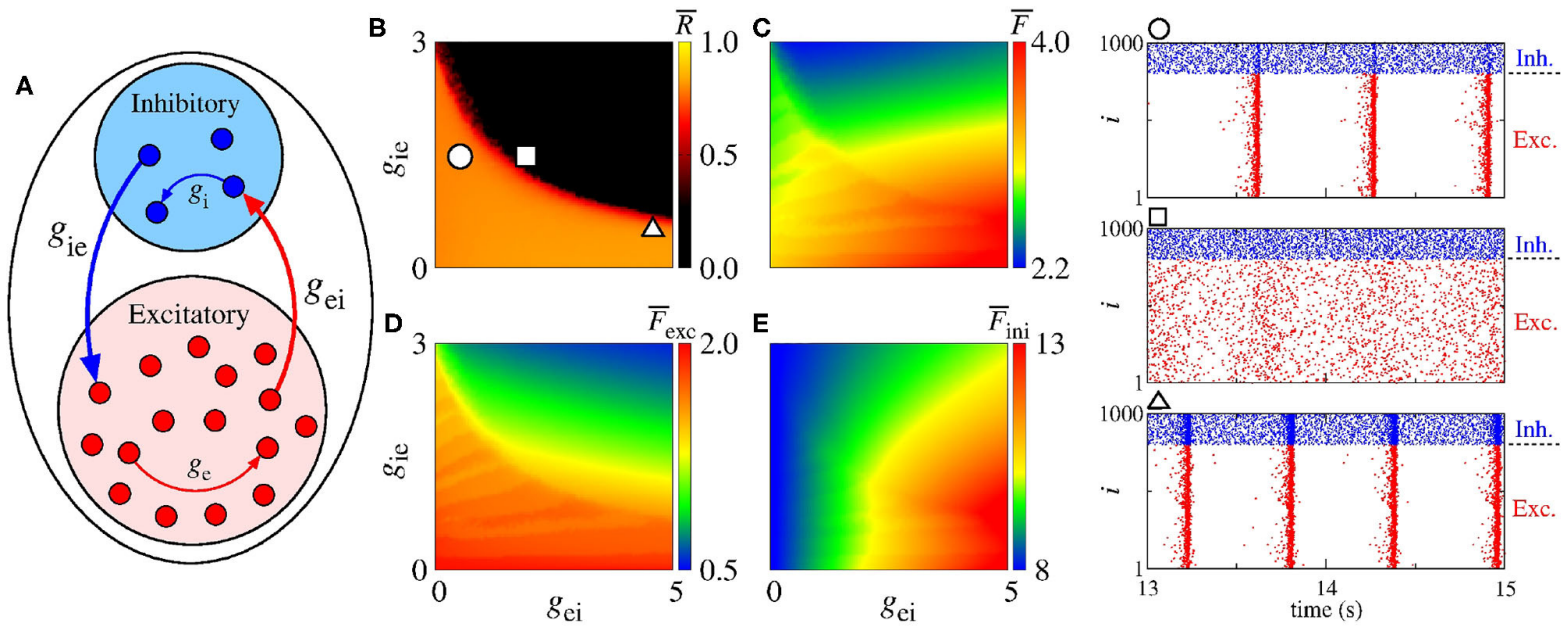
**(A)** Schematic representation of a neural network with a mix of excitatory and inhibitory neurons without autapses. Parameter spaces *g*_ei_ × *g*_ie_ for *g*_e_ = 0.5 nS and *g*_i_ = 2 nS, where the color bars correspond to **(B)** $\bar{R}$, **(C)** $\bar{F}$, **(D)** ${\bar{F}}_{\text{exc}}$, and **(E)** ${\bar{F}}_{\text{inh}}$. The circle, square, and triangle symbols in **(B)** represent the values of the parameters considered in the computation of the raster plots shown in the right side. The blue and red points in the raster plots indicate the firing of the inhibitory and excitatory neurons over time, respectively.

Desynchronous neural activities in balanced excitatory/inhibitory regimes have been reported in Borges et al. (2020) and Ostojic (2014). Based on these results, here we study different combinations of *g*_e_, *g*_i_, $g_{\text{e}}^{\text{aut}}$, and $g_{\text{i}}^{\text{aut}}$ values in the parameter space *g*_ei_ × *g*_ie_ (see Figure 7). The existence of synchronous and desynchronous activities depend on the values of these parameters which are related to the conductances. We focus on a set of parameters for which synchronous activities appear. Firstly, we consider *g*_e_ = 0.5 nS and *g*_i_ = 2 nS in a neural network without autaptic connections.

Figure 7A shows a schematic representation of excitatory (red circles) and inhibitory (blue circles) neurons, where *g*_ei_ (*g*_ie_) correspond to the conductance from excitatory to inhibitory (from inhibitory to excitatory) neurons in the absence of autapses. Figure 7B presents the mean order parameter ($\bar{R}$) and the circle, square and triangle symbols indicate the values of the parameters considered in the computation of the raster plots shown in the right hand-side. The values of the conductances used to compute the raster plots are given by *g*_ei_ = 0.5 nS and *g*_ie_ = 1.5 nS for the circle, *g*_ei_ = 1.8 nS and *g*_ie_ = 1.5 nS for the square, and *g*_ei_ = 4.5 nS and *g*_ie_ = 0.5 nS for the triangle symbols. The blue and red points in the raster plots represent the firing of the inhibitory and excitatory neurons over time, respectively. Kada et al. (2016) reported that synchronization can be suppressed by means of inhibitory to excitatory or excitatory to inhibitory connection heterogeneity. Here, we observe that a minimal interaction between the excitatory and inhibitory neurons is required to suppress high synchronous patterns. In Figure 7C, we verify that $\bar{F}$ decreases when *g*_ie_ increases. Figures 7D,E show that ${\bar{F}}_{\text{exc}}$ and ${\bar{F}}_{\text{ini}}$ can decrease when *g*_ie_ increases. In addition, ${\bar{F}}_{\text{exc}}$ decreases and ${\bar{F}}_{\text{ini}}$ increases when *g*_ei_ increases. When the neural populations are uncoupled (*g*_ei_ = *g*_ie_ = 0), the firing rate difference in the excitatory and inhibitory neurons are mainly due to the adaptation properties of these cells.

**Figure 8:**
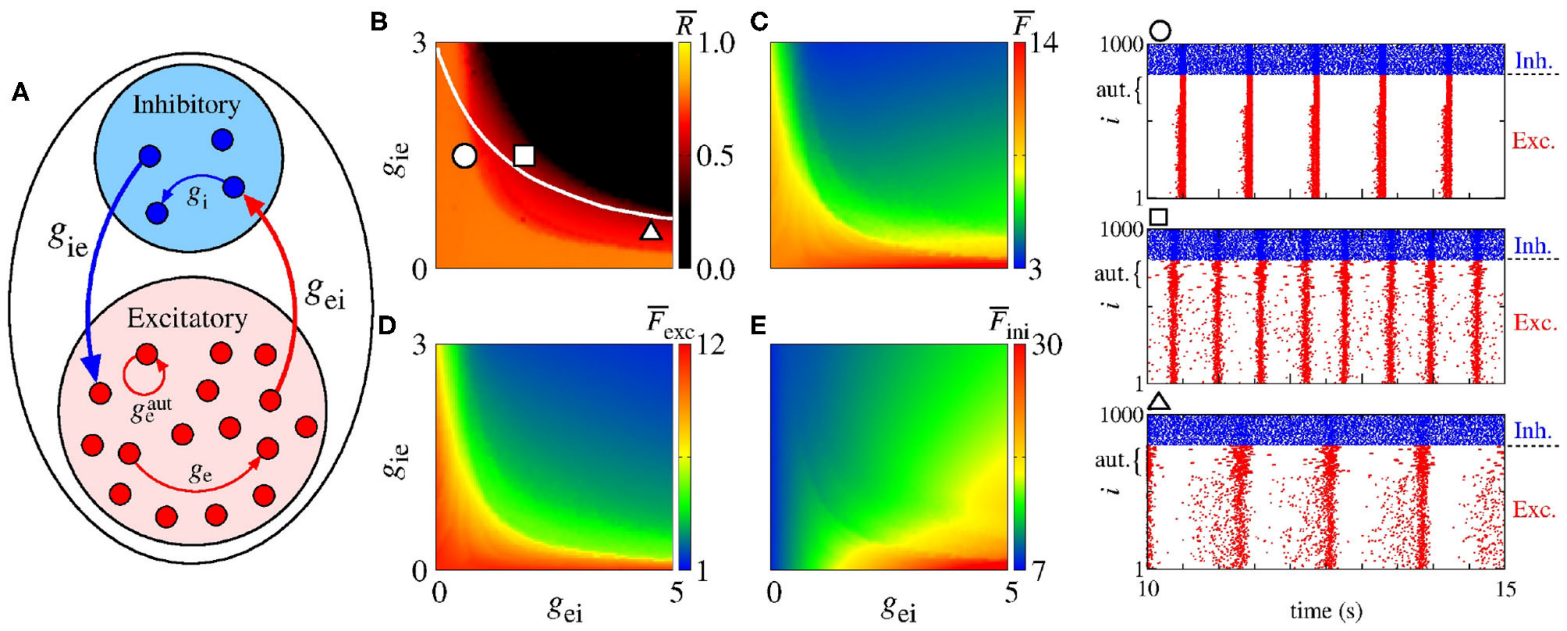
**(A)** Schematic representation of a neural network with a mix of excitatory and inhibitory neurons with excitatory autapses. Parameter spaces *g*_ei_ × *g*_ie_ for *g*_e_ = 0.5 nS, *g*_i_ = 2 nS and $g_{\text{e}}^{\text{aut}}=30$ nS, where the color bars correspond to **(B)** $\bar{R}$, **(C)** $\bar{F}$, **(D)** ${\bar{F}}_{\text{exc}}$, and **(E)** ${\bar{F}}_{\text{inh}}$. The circle, square, and triangle symbols in **(B)** represent the values of the parameters considered in the computation of the raster plots shown in the right side. The blue and red points in the raster plots indicate the firing of the inhibitory and excitatory neurons over time, respectively. The curly brackets in the upper left corner of the plots denote the autaptic neurons considered.

Figure 8A shows a schematic representation of a network with a mix of excitatory and inhibitory neurons in the presence of excitatory autapses. In Figure 8B, we present the parameter space *g*_ei_ × *g*_ie_ for $g_{\text{e}}^{\text{aut}}=30$ nS, where the color bar corresponds to $\bar{R}$. The white solid line in the parameter space indicates the transition from desynchronous to synchronous behavior in the network without excitatory autaptic conductance ($g_{\text{e}}^{\text{aut}}=0$), as shown in Figure 7B. The raster plots in the right hand-side of the figure are computed using the values of the parameters indicated by the circle, square, and triangle symbols in Figure 7B. In Figures 8C–E, we see that excitatory autapses can increase the firing rate of all neurons, changing the mean firing rate dependence on *g*_ei_ and *g*_ie_.

## 4. Conclusions

In this paper, we investigated the influence of autapses on neural synchronization in networks of coupled adaptive exponential integrate-and-fire neurons. Depending on the parameters of the system, the AEIF model exhibits spike or burst activity. In our simulations, we considered neurons randomly connected with chemical synapses in the absence or presence of autapses.

We verified that the type of synaptic connectivity plays a different role in the dynamics in the neural network, especially with regard to synchronization. It has been reported that excitatory synapses promote synchronization and firing pattern transitions. In our simulations, we found that excitatory autapses can generate firing pattern transitions for low excitatory synaptic conductances. The excitatory autaptic connections can promote desynchronization of all neurons or only of the autaptic ones in a network with neurons initially synchronized. The excitatory autapses can also increase the firing rate of all neurons. In a network with only inhibitory synapses, we did not observe inhibitory synapses and autapses promoting synchronization. We saw a reduction and increase of the firing rate of the autaptic and non-autaptic neurons, respectively, due to inhibitory autapses.

Finally, in a network with a mix of excitatory and inhibitory neurons, we saw that the interactions among the populations are essential to avoid high synchronous behavior. The excitatory to inhibitory synaptic connectivities promote the increase (decrease) of the firing rate of the inhibitory (excitatory) populations. On the other hand, the inhibitory to excitatory synaptic connectivities give rise to the decrease of the firing rate of both populations. We observed that the excitatory autapses can reduce the synchronous activities, as well as induce neural synchronization. For small conductances, excitatory autapses can not change synchronization significantly. Consequently, our results provide evidence on the synchronous and desynchronous activities that emerge in random neural networks with chemical, inhibitory and excitatory, synapses where some neurons are equipped with autapses.

In a more general context, the role of network structure upon synchronicity in networks with delayed coupling and delayed feedback was studied, and very general classifications of the network topology for large delay were given by Flunkert et al. (2010, 2014), e.g., it was shown that adding time-delayed feedback loops to a unidirectionally coupled ring enables stabilization of the chaotic synchronization, since it changes the network class. We believe that the absence or presence of autapses has similar effects upon synchronization. In future works, we plan to compute the master stability function of networks with autapses to compare with the stability of synchronization in delay-coupled networks.

## Data Availability Statement

The raw data supporting the conclusions of this article will be made available by the authors, without undue reservation.

## Author Contributions

PRP and KCI designed the work, developed the theory, and performed the numerical simulations. AMB wrote the manuscript with support from ILC and JK. The authors revised the manuscript several times and gave promising suggestions. All authors also contributed to manuscript revision, read, and approved the submitted version.

## Conflict of Interest

The authors declare that the research was conducted in the absence of any commercial or financial relationships that could be construed as a potential conflict of interest.
